# Evidence that large vessels do affect near infrared spectroscopy

**DOI:** 10.1038/s41598-022-05863-y

**Published:** 2022-02-09

**Authors:** Stefano Seddone, Leonardo Ermini, Piero Policastro, Luca Mesin, Silvestro Roatta

**Affiliations:** 1 Department of Neuroscience, Università degli Studi di Torino, Turin, Italy; 2 Department of Electronics and Telecommunications, Politecnico di Torino, Turin, Italy

**Keywords:** Blood flow, Vasodilation, Near-infrared spectroscopy

## Abstract

The influence of large vessels on near infrared spectroscopy (NIRS) measurement is generally considered negligible. Aim of this study is to test the hypothesis that changes in the vessel size, by varying the amount of absorbed NIR light, could profoundly affect NIRS blood volume indexes. Changes in haemoglobin concentration (tHb) and in tissue haemoglobin index (THI) were monitored over the basilic vein (BV) and over the biceps muscle belly, in 11 subjects (7 M – 4 F; age 31 ± 8 year) with simultaneous ultrasound monitoring of BV size. The arm was subjected to venous occlusion, according to two pressure profiles: slow (from 0 to 60 mmHg in 135 s) and rapid (0 to 40 mmHg maintained for 30 s). Both tHb and THI detected a larger blood volume increase (1.7 to 4 fold; p < 0.01) and exhibited a faster increase and a greater convexity on the BV than on the muscle. In addition, NIRS signals from BV exhibited higher correlation with changes in BV size than from muscle (r = 0.91 vs 0.55, p < 0.001 for THI). A collection of individual relevant recordings is also included. These results challenge the long-standing belief that the NIRS measurement is unaffected by large vessels and support the concept that large veins may be a major determinant of blood volume changes in multiple experimental conditions.

## Introduction

Near-Infrared Spectroscopy (NIRS) has been extensively applied for the assessment of changes in tissue oxygenation and blood volume in skeletal muscles due to its non-invasiveness, its ability to operate in real-time and for being little affected by movement artefacts^1–3^. Briefly, the functioning of NIRS depends on the emission of a NIR light radiation from the emitting optode which travels through the underlying tissues and gets either absorbed or scattered, the backscattered fraction being collected by the receiving optode. Depending on the amount of returning light at different wavelengths, NIRS devices can assess changes in the concentration of Oxy-(Haemoglobin + Myoglobin) and Deoxy-(Haemoglobin + Myoglobin) (O_2_Hb/HHb) thus providing indications on tissue oxygenation and blood volume.

**Figure 1:**
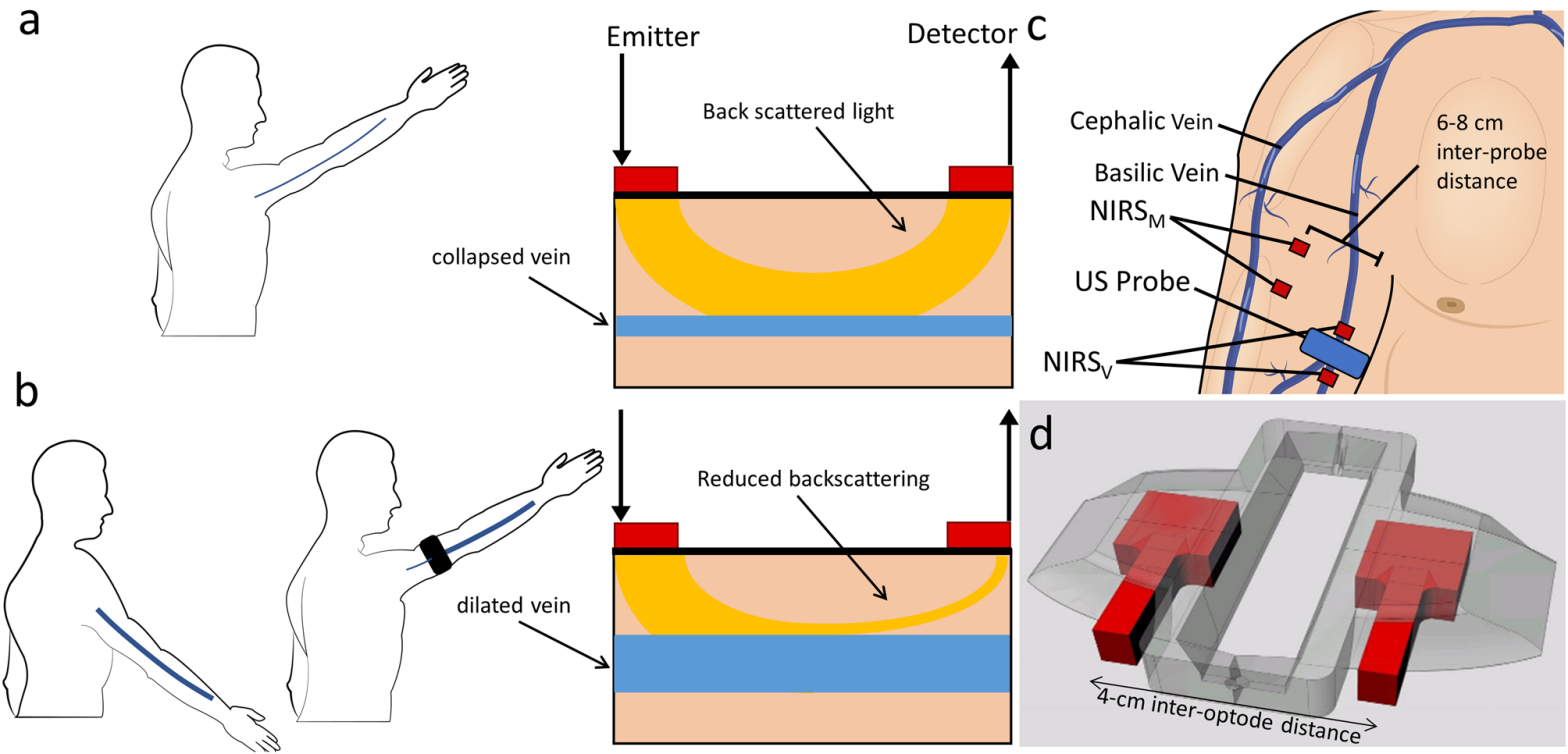
(**a**) Vein collapse during arm elevation results in little absorption of NIR light by the vein and consequently in high intensity of the light backscattered to the detector. (**b**) Vein dilation, during, e.g., arm lowering or venous occlusion, increases the absorption and reduces the amount of backscattered light, which produces an increase of NIRS blood volume indexes; (**c**) location of NIRS probes: one NIRS probe was positioned over the biceps muscle belly (NIRS_M_) and one over the basilic vein (NIRS_V_); an ultrasound probe was coupled with NIRS_V_ to monitor size changes in the cross-sectional area of the basilic vein; (**d**) custom 3D-printed probe holder, designed to embed the linear US-probe (not drawn) in-between NIRS optodes (in red). The figure was created with Adobe Illustrator 2020, v. 24.1.1, www.adobe.com (**a,c**), MS PowerPoint 2019, v. 2111, www.microsoft.com (**b**), and Rhino 7 v.7.13, www.rhino3d.com (**d**).

The interpretation of NIRS signals is complicated by several limitations of the methodology, among which the inability to discriminate between haemoglobin and myoglobin, the difficulty to discriminate between superficial and deep tissues, the need to estimate or assess the actual optical pathlength of NIRS light, and the uncertainty about the actual vessels contributing to the measurement, with regard to type (arterial/capillary/venous) and size^4^. As for vessel size, it is generally assumed that only vessels smaller than 1-mm diameter contribute to the NIRS signal^4–6^. This assumption appears to stem from a consideration originally expressed by Mancini et al.^7^ that the large vessels would completely absorb the incident light and preclude backscattering, due to the high heme concentration. Therefore, the collected light would arise mostly from small vessels, such as capillaries^6^, arterioles, and venules^8,9^. However, we believe that this reasoning is correct only as long as the size of the large vessels does not change. In fact, an increase in vessel size would increase the amount of absorbed light and decrease the fraction of backscattered light, as described in Fig. 1a,b. The possibility that large vessels may change their size is particularly relevant for the highly compliant veins, which frequently exhibits large size changes in response to changes in intravascular and extravascular pressure, e.g., the vein size in a limb increases when the limb is moved from an independent (Fig. 1a) to a dependent position (Fig. 1b), due to increased hydrostatic blood pressure or may decrease due to the external compressions produced by muscle contractions. However, due to the consideration anticipated above, the possibility that large vessels may actually affect NIRS measurements in skeletal muscles is generally neglected, despite the issue has never been specifically addressed or experimentally verified. On the contrary, contamination of NIRS signals by superficial visible veins has been occasionally evidenced^10,11^.

Aim of the present study was to test the hypothesis that large vessels may significantly affect NIRS signals. To this aim, NIRS monitoring was conducted in the biceps muscle, one probe being located over a vessel-free area and the other one over the basilic vein (BV). In order to allow for continuous monitoring of the BV cross-section by ultrasound imaging, that could be problematic during postural changes or exercise, repeatable changes in BV size were obtained in static conditions by controlled venous occlusion, proximally delivered. Evidences in support of the hypothesis were collected both from average responses to venous occlusions as well as from relevant observations derived from individual recordings.

## Methods

Eleven healthy subjects were recruited in this study (31 ± 8 year—7 M 4F). The study was approved by the Bioethics Committee of the University of Torino, no diagnosed diseases were reported by any of the participants. All the experiments were performed in accordance with Declaration of Helsinki, each subject signed the informed consent form.

### Subject setting

The subjects were seated on a comfortable and adjustable chair with the back supported, in a quiet room at a constant temperature of ~ 21–22° C. The left arm was elevated until reaching ~ 135° of shoulder flexion and passively maintained supine over a padded rigid support. A gentle bandage at the wrist served to secure and stabilize the arm position. This arrangement produced a collapse of the basilic vein in most subjects, due to the decrease in venous blood pressure according to hydrostatic gradients.

### Venous occlusion

Controlled pneumatic compressions were elicited by the inflation of a pneumatic paediatric cuff (Gima, Gessate, Italy; width 5 cm), close to the armpit. The cuff inflation was operated by two solenoid valves (VXE2330-02F-6D01, SMC, Tokyo, Japan), granting rapid in-flow and out-flow, and a proportional valve (ITV0010, SMC, Tokyo, Japan), granting precise pressure maintenance at the desired level. Compressed air (1 bar) was provided by an air compressor, and the Spike2 software (version 9, Cambridge Electronic Design, Cambridge, UK) coupled with an I/O board (CED Micro 1041, Cambridge Electronic Design, Cambridge, UK) was used to drive the above-mentioned valves to implement both rapid and slow inflation/deflation patterns^12–14^.

### NIRS measurements

NIRS measurements were performed using a continuous wave spectrometer (NIRO200NX, Hamamatsu Photonics, Hamamatsu City, Japan), implementing the modified Beer–Lambert (BL) and spatially resolved spectroscopy (SRS) methods^15^. The former allows to detect changes in oxygenated (O_2_Hb) and deoxygenated haemoglobin concentrations (HHb) expressed in μmol/L, assuming a *differential path-length factor* of 3.6^16^. The sum of these two components, tHb = O_2_Hb + HHb, indicate changes in total blood volumes, with respect to a basal reference level, set to 0. The SRS technique provides an additional blood volume indicator, the tissue haemoglobin index (THI), which yields the relative change in haemoglobin concentration (dimensionless) with respect to the beginning of the recording, arbitrarily set to 1. Notably, NIRS spectrometers are unable to discriminate between haemoglobin and cytoplasmic myoglobin, therefore all measurements are actually referred to the sum of the two components (haemoglobin + myoglobin) in the sample volume.

Two NIRS measurements were simultaneously performed. One probe was positioned at the distal third of the arm, on the medial side, just over the BV and more laterally on the biceps brachii muscle, over an area virtually free of large vessels (Fig. 1c). Echographic guidance allowed for precise identification of the BV, whose orientation was marked with a marker on the overlying skin. A custom 3D-printed holder was designed to accommodate both the NIRS optodes (inter-optode distance of 4 cm; Fig. 1d), and a linear US probe (see below) in between and transversally to the NIRS probe, so that the NIRS probe could be oriented parallel to the BV while the US probe could be used to display and record its cross-sectional area. The NIRS optodes position over the biceps muscle belly were housed in their native probe holder (same inter-optode distance of 4 cm). The NIRS probes were sufficiently separated from each other (6–8 cm) to avoid interference and were stuck to the skin by double-sided medical adhesive tape and further secured by additional overlying tape. The 4-cm rather than the 3-cm inter-optode distance was chosen because it grants a better rejection of the interference from the cutaneous circulation (for the SRS parameter, THI) and increases the depth of the sample volume, considered to be about half of the inter-optode distance.

### Ultrasound imaging

The cross-section of the BV was continuously monitored by B-mode US imaging (MyLab 15, Esaote S.p.A., Genoa, Italy) using a linear array (LA 523, Esaote, Genoa, Italy) oriented transversally to the vessel lumen at an insonation angle of ~ 90° (US frequency of 12 MHz). The US probe was maintained firmly in place by a mechanical arm, anchored to the same support holding the subject’s arm^12^. From the B-mode scan, the thickness of cutaneous and subcutaneous tissue layer could be measured in all subjects.

### Experimental protocol

After stabilization of hemodynamic signals, a randomized sequence of venous occlusions was performed according to the following pressure profiles: (i) a slow inflation from 0 to 60 mmHg in 135 s followed by a plateau, 60 mmHg for 30 s, followed by slow deflation 60–0 mmHg in 135 s; (ii) rapid inflation to 40 mmHg, achieved in 2 s, maintained for 30 s and passively deflated in about 2 s. The different occlusions were separated by at least 30 s. Other occlusion patterns or pressure levels were occasionally tested but were not systematically analysed. Venous occlusion led to a gradual rise of blood pressure in distal veins and the ensuing increase in BV size could be recorded by US imaging.

### Data acquisition and processing

The NIRS signals were digitally acquired at 50 Hz along with the cuff pneumatic pressure (CED Micro 1041, Cambridge Electronic Design, Cambridge, United Kingdom) and stored for later analysis on the computer using Spike2 software (version 9.04b, Cambridge Electronic Design, Cambridge, United Kingdom). The analysis was limited to the blood volume indicators tHb and THI, collected from the probe positioned over the basilic vein (tHb_V_ and THI_V_) and over the biceps muscle belly (tHb_M_ and THI_M_). Responses to venous occlusion were characterized in terms of (1) maximum change in blood volume, as expressed by the peak value of the recordings (considering that basal, i.e., pre-occlusion levels for tHb and THI are 0 and 1, respectively); (2) convexity of the rising volume curve, quantified as the area comprised between the normalized curve and the virtual linear trend, divided by the duration of the rising phase (nAUC) so that nAUC is dimensionless and equals 0.5 for maximum convexity, 0 for a rising linear trend and − 0.5 for maximum concavity. The area is calculated over the whole rising phase, defined as the time interval between the moment at which the pressure exceeded 10 mmHg and the moment at which the blood volume reaches its maximum value (i.e. at the end of the plateau), while the normalization of the curve was achieved by subtracting the minimum to the original curve and subsequently dividing the resulting curve by its current maximum value. In this way the normalized rising volume curve had its minimum value at 0 and its maximum value at 1.

Volume-pressure curves were constructed by quadratic fitting of the average tHb and THI response to slow cuff deflation (from 60 to 0 mmHg in 135 s, described above). Compliance curves were then obtained as first analytical derivatives of the volume-pressure curves^17^.

Video clips of US imaging of the BV, lasting for the whole duration of the venous occlusions, were recorded and later analysed by a custom image processing software, originally developed for the inferior vena cava^18,19^, capable of tracking the vein borders, and reporting the time course of the vessel cross-sectional area during cuff inflation. Proper detection of vein borders was validated by visual inspection of the video clips with superimposed vessel reconstruction. Automatic vessel detection could fail due to poor quality of the US imaging or due to the collapse of the vein. In these cases, data were either discarded or integrated with manual measurements.

### Statistical analysis

Comparisons between responses of NIRS signals from the two probes (vein vs. muscle), in terms of magnitude, latency and convexity were done using a non-parametric paired Wilcoxon T test. Similarity of the response of NIRS variables with changes in BV size, for each subject, was quantified by the Pearson’s correlation coefficient, then Fisher transformation was applied to those ρ values, and finally each distribution was tested using a non-parametric Wilcoxon T test. The procedure was performed for both tHb and THI and for both slow and rapid venous occlusion profiles.

## Results

In two subjects, THI_V_ saturated during venous occlusion. The measurements were then discarded and repeated after slightly displacing the NIRS probe by few millimetres. This problem was not encountered in signals collected from the muscle belly.

In the resting condition, the BV collapsed in most subjects. Average BV depth (centre of the vessel) was 10.2 ± 4.2 mm, extending from 7.4 ± 4.2 to 13.1 ± 4.2 mm at its maximum dilatation at the plateau of slow ramp occlusions. The thickness of cutaneous and subcutaneous tissue layers was assessed from the ultrasound scans and found to be 2.1 ± 0.3 mm*.*

### Slow vein occlusions

**Figure 2:**
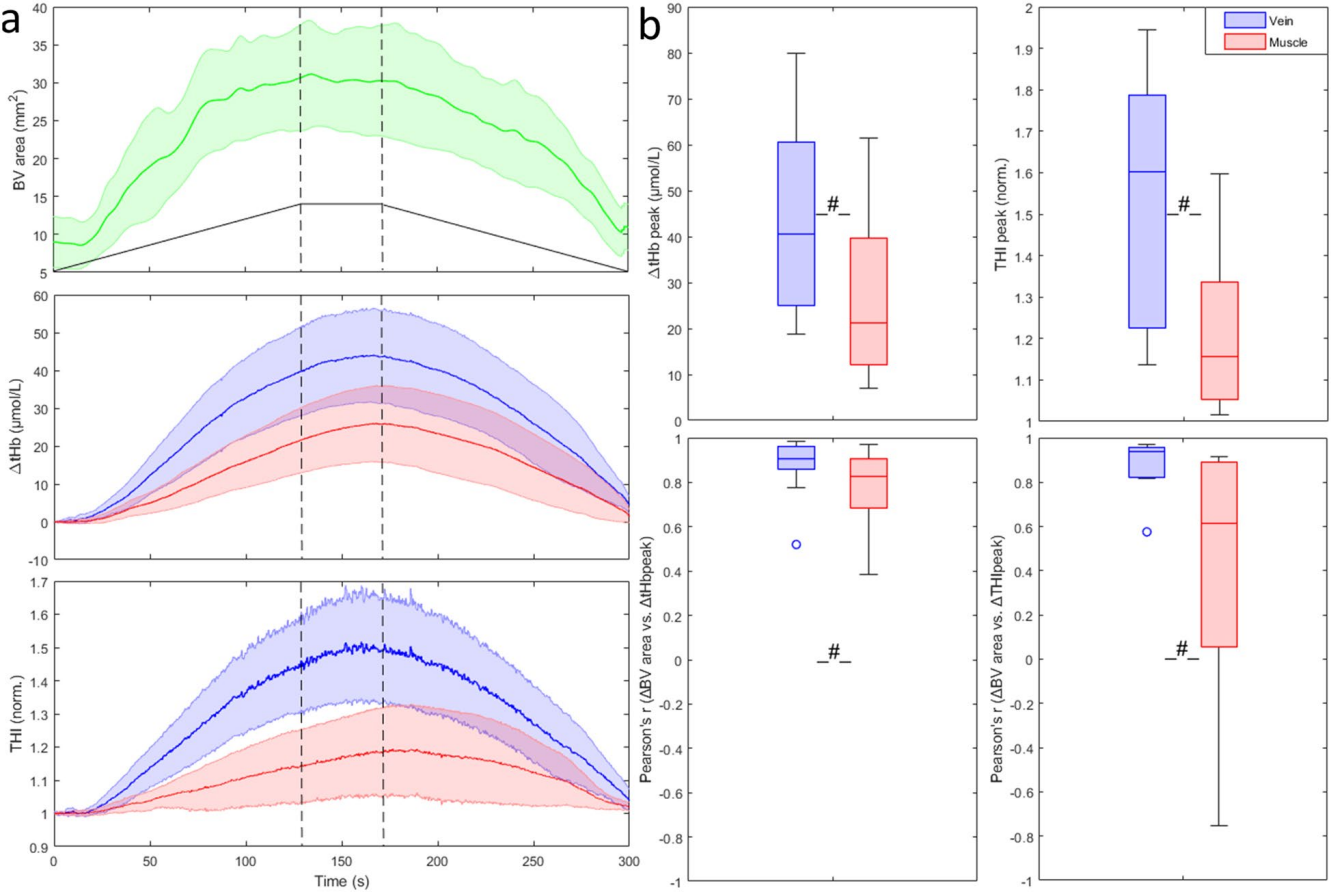
(**a**) Average response to slow venous occlusion of the cross-sectional area of the basilic vein (green) and of tHb and THI as detected on the muscle (red) and on the vein (blue). Shaded areas represent the 95% confidence interval of the mean. The black trapezoid depicted in the top graph qualitatively reproduces the cuff pressure during inflation to 60 mmHg, plateau and subsequent deflation to 0 mmHg, while the vertical dashed lines indicate end of inflation (first one) and beginning of deflation (second one). (**b**) Peak response of blood volume indexes, collected from vein and muscle probes (top), Pearson’s coefficient of the correlation between changes in BV cross-sectional area and in blood volume indices (bottom). *p < 0.05; ^#^p < 0.01.

The slow venous occlusion effectively increased the BV cross-sectional area from 8.9 ± 5.7 to 32.7 ± 12.1 mm^2^ and produced a slow increase, followed by a slow decrease in blood volume indexes as shown in Fig. 2a. Notably, the indexes exhibited systematically larger changes over the BV than over the muscle belly, on average: ΔtHb_V_ = 44.8 ± 21.3 μmol/L vs. ΔtHb_M_ = 27.1 ± 17.6 μmol/L (p < 0.01) and THI_V_ = 54 ± 31% vs. THI_M_ = 22 ± 21% (p < 0.002) (Fig. 2b).

The time course of the responses was also slightly different between the two measurement areas: indeed, tHb_V_ exhibited a more rapid increase as compared to tHb_M_ as shown by the higher curvature nAUC (0.052 ± 0.076 vs. − 0.005 ± 0.045, p = 0.03). For THI the differences did not reach statistical significance. However, it is worth to report that in 3 subjects THI_M_ did not increase or slightly decreased.

Both tHb_V_ and THI_V_ values were better correlated to BV size than the corresponding muscle indexes (p < 0.001): tHb_V_ ρ = 0.92, THI_V_ ρ = 0.91, tHb_M_ ρ = 0.83, THI_M_ ρ = 0.55 (Fig. 2b).

**Figure 3:**
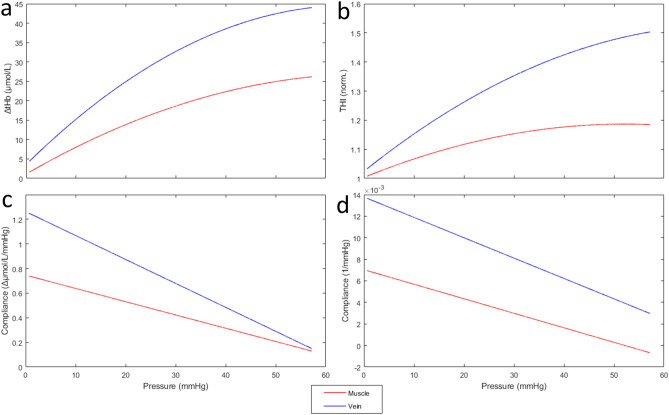
Volume-pressure curves, obtained by 2° order polynomial fitting of average responses to slow cuff-deflation of Fig. 2a (top), and compliance curves, obtained as the slopes of the corresponding volume-pressure curves (bottom), for tHb (left) and THI (right) collected from vein (blue) and muscle (red). Note the large difference in the compliance estimated from vein and muscle.

Volume-pressure and vascular compliance curves were constructed from the average tHb and THI curves of Fig. 2, collected from vein and muscle during cuff deflation (Fig. 3). Note that the compliance estimated from the probe on the vein is considerably higher than from the muscle.

### Rapid vein occlusions

**Figure 4:**
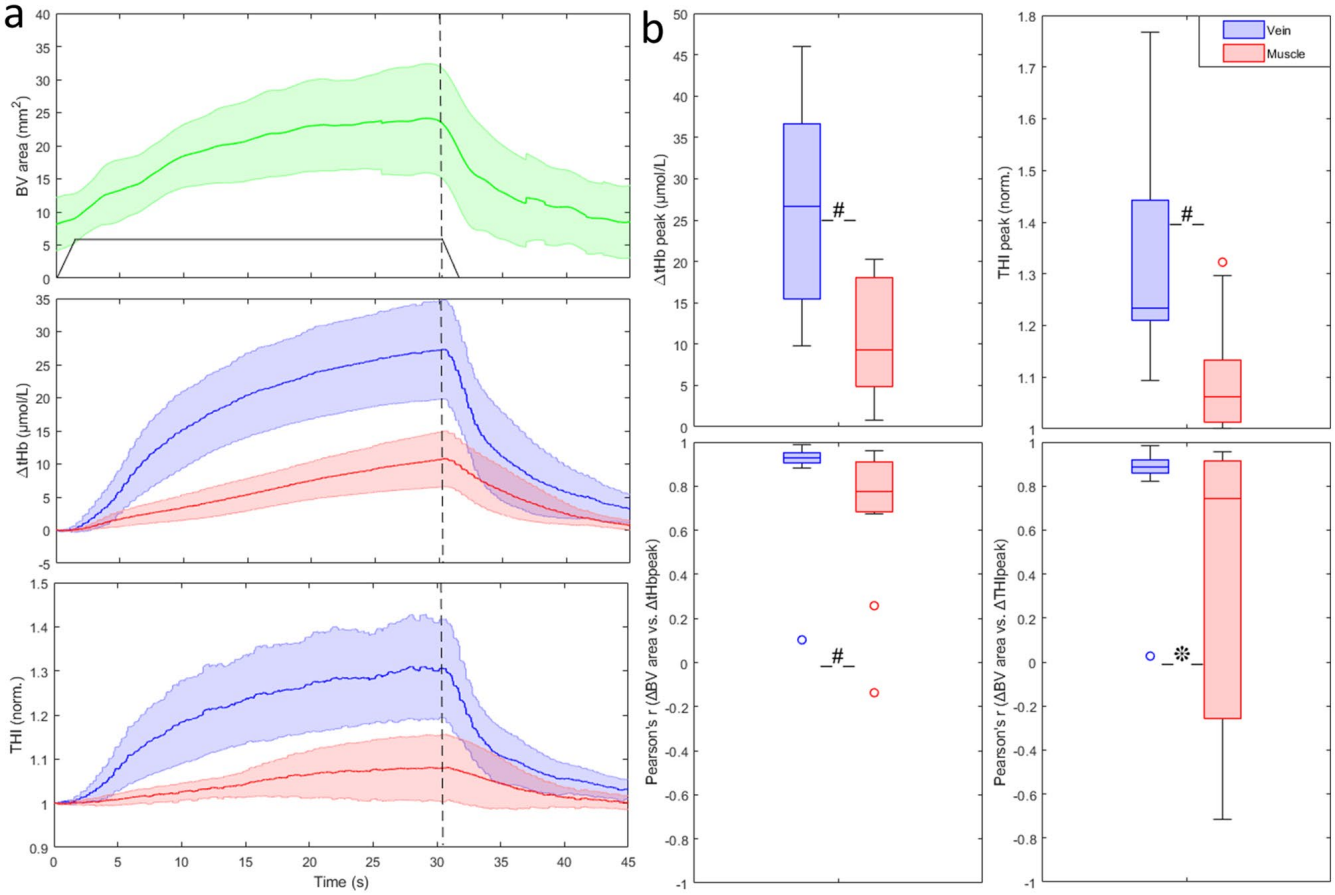
Average response to rapid venous occlusion of the cross-sectional area of the basilic vein (green) and of tHb and THI as detected on the muscle (red) and on the vein (blue). Shaded areas represent the 95% confidence interval of the mean. The black trapezoid depicted in the top graph qualitatively reproduces the cuff pressure during inflation to 40 mmHg, plateau and subsequent deflation to 0 mmHg, while the vertical dashed line indicates the beginning of deflation. (**b**) Peak response of blood volume indexes, collected from vein and muscle probes (top), Pearson’s coefficient of the correlation between changes in BV cross-sectional area and in blood volume indices (bottom). *p < 0.05; ^#^p < 0.01.

As described for slow-ramp occlusions, rapid occlusions also produced an increase in BV cross-sectional area from 8.8 ± 6.5 to 25.5 ± 13.4 mm^2^, and relevant increase in blood volume indexes (Fig. 4a) with significantly larger changes in the vein than in the muscle: ΔtHb_V_ = 27.3 ± 12.6 μmol/L vs. ΔtHb_M_ = 11 ± 7.1 μmol/L (p < 0.002) and THI_V_ = 32 ± 21% vs. THI_M_ = 8 ± 9% (p < 0.001) (Fig. 4b). A clear-cut difference in time course between vein and muscle is here significantly achieved for tHb and THI, both exhibiting a higher convexity during the rising phase of blood volume (tHb: 0.18 ± 0.08 vs. 0.07 ± 0.10, p < 0.05; for THI: 0.16 ± 0.07 vs. 0.06 ± 0.12, p < 0.05), on the vein as compared to the muscle. Also in this case 4/11 subjects exhibited no change or slight decrease in THI_M_.

As shown in Fig. 4b, vein indexes were generally better correlated to BV cross-sectional area than muscle indexes: average Pearson’s ρ was 0.92 vs. 0.78 for tHb (p < 0.01) and 0.89 vs. 0.56 for THI (p < 0.05) in vein vs. muscle.

### Single relevant cases

In addition to the afore-described differences in the NIRS signals averaged over all subjects, few relevant examples of individual recordings are presented below, further evidencing peculiar aspects of the influence that large vessels exert on NIRS signals.

**Figure 5:**
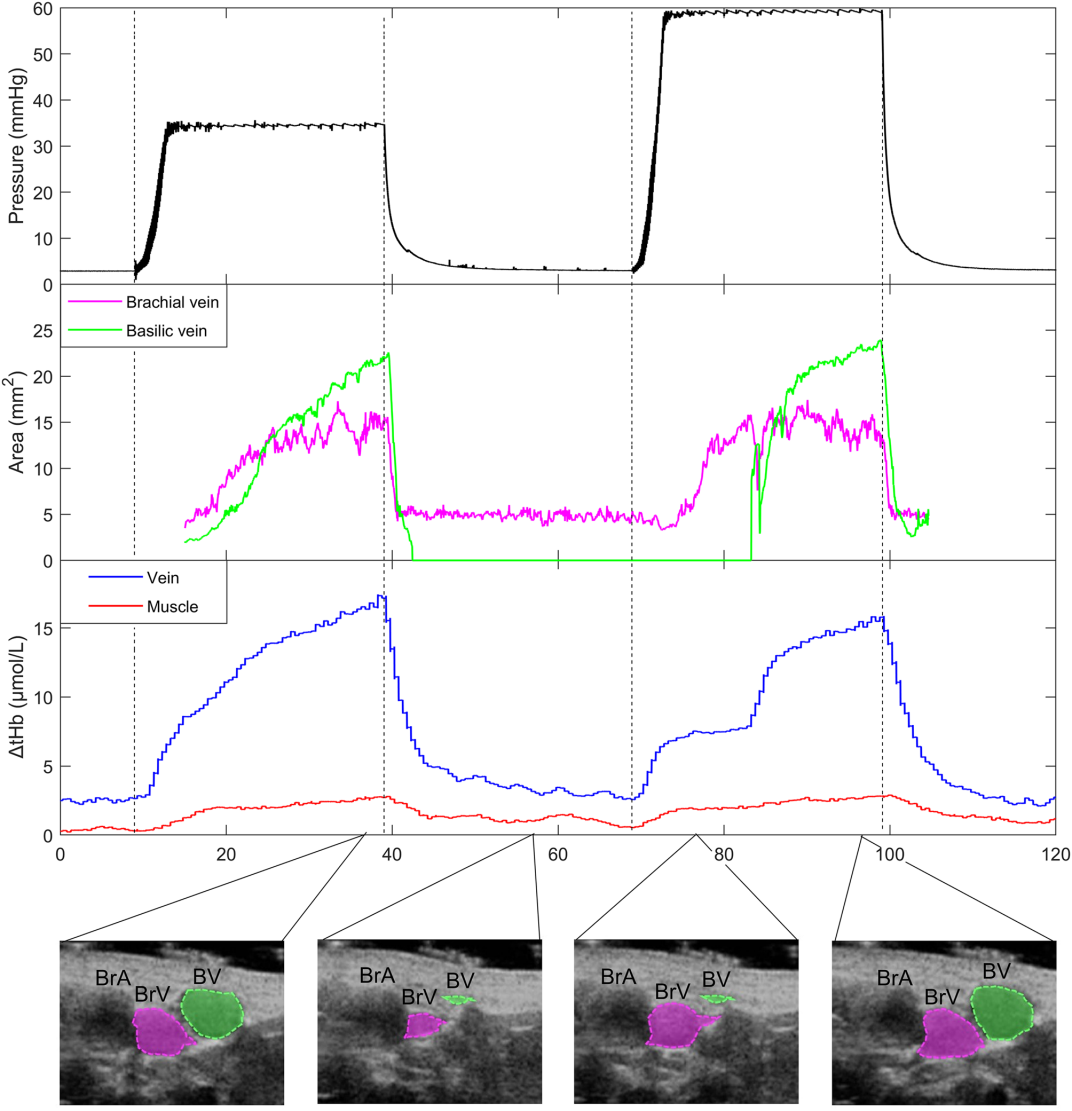
Original traces from a single subject showing the responses to two subsequent short lasting venous occlusions (30 and 60 mmHg, respectively). From top to bottom: cuff pressure; Cross-sectional area of brachial vein and basilic vein; tHb signals from the vein (blue) and muscle (red) probes. At the bottom, single frames of US imaging of blood vessels underneath the NIRS_V_ probe (as indicated in Fig. 1b) are displayed, with superimposed coloured shadings indicating the extent of collapse/dilatation of the brachial (BrV) and basilic (BV) veins. The brachial artery (BrA) is also indicated. Note the unusual 2-steps increase in tHb_V_ in response to the second occlusion, which is explained by the delayed dilatation of the basilic vein. The fact that the increase in venous size is delayed, compared to tHb_V_ is due to the NIRS sample volume extending proximally to the US insonation site (during venous occlusion, venous pressure increases earlier at proximal than at distal sites, in the raised arm). Vertical dashed lines indicate start of cuff inflation and deflation.

**Figure 6:**
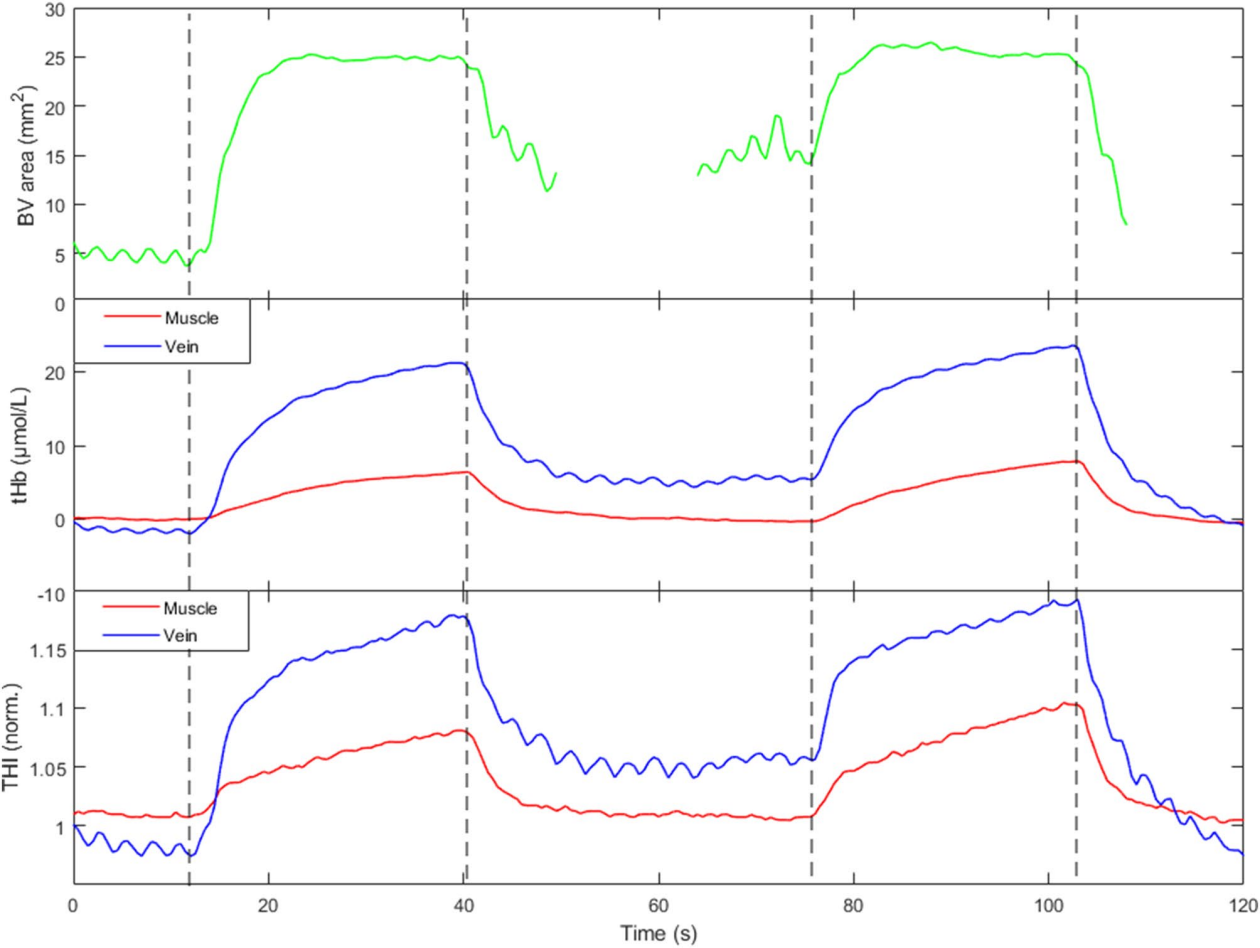
Original traces from a single subject showing the responses to two subsequent short lasting occluding stimuli (both at 40 mmHg). From top to bottom: cross-sectional area of the basilic vein (green), THI, and tHb signals collected from the vein (blue) and from the muscle (red). Note the presence of an oscillatory component of respiratory origin, synchronously appearing on the vessel cross-sectional area and on the venous NIRS signals only. Vertical dashed lines indicate start of cuff inflation and deflation.

**Figure 7:**
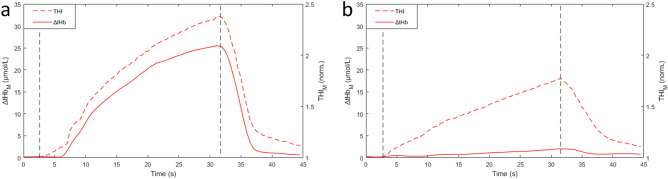
Original traces from the same subject showing tHb and THI responses to 40-mmHg venous occlusions subsequently recorded on the biceps brachii muscle belly at the original site (**a**) and after lateral displacement of 1 cm (**b**). Note the different response of THI, attributed to a small vein in the sample volume in (**b**). Vertical dashed lines indicate start of cuff inflation and deflation.

**Figure 8:**
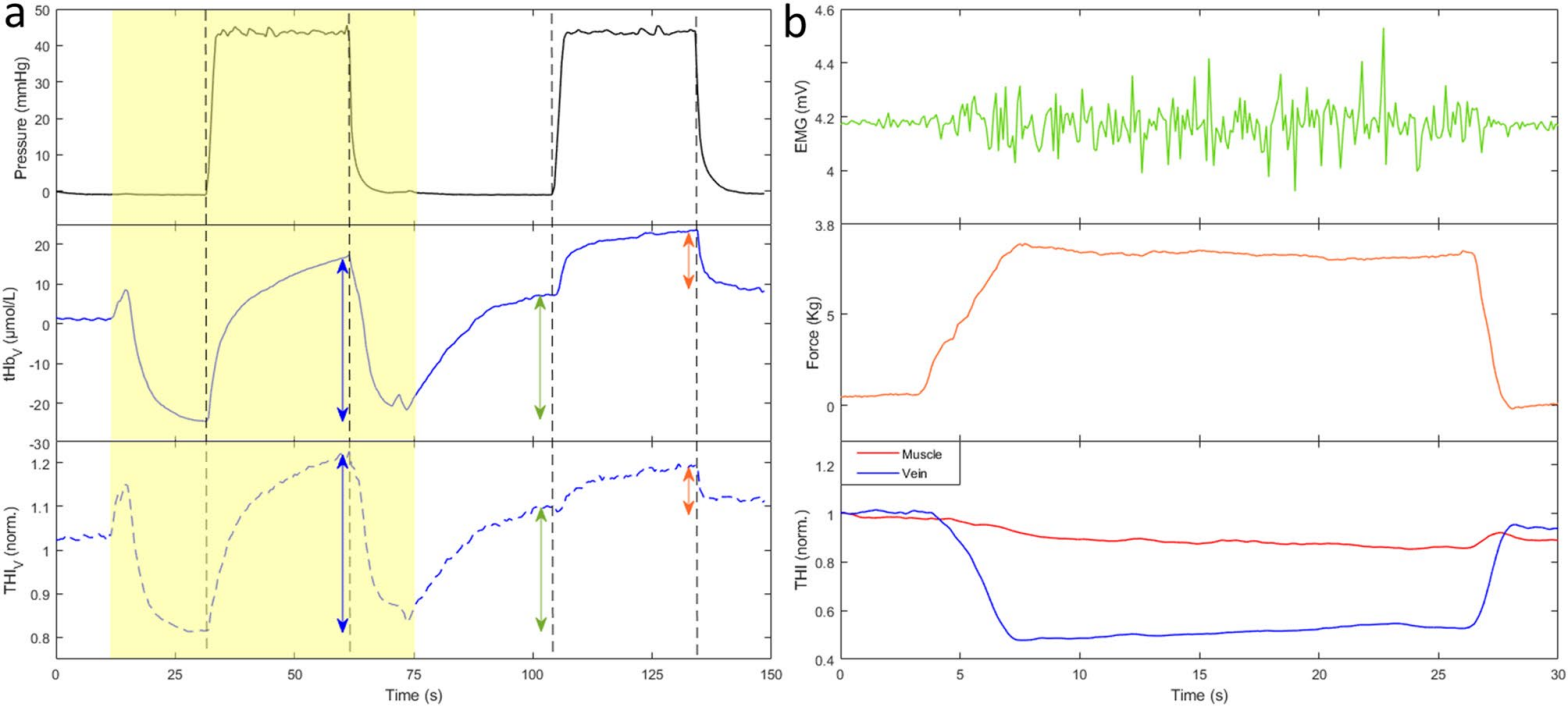
(**a**) Effect of postural changes on blood volume indices in a representative recording. From top to bottom, cuff pressure, tHb_V_ and THI_V_ collected from a single NIRS probe placed over the basilic vein (same set-up of Fig. 1b). The arm was moved from below to above (yellow-shaded area) and again returned to below heart level. Proximal venous occlusion (40 mmHg) was also performed for comparison, as indicated by the cuff pressure signal. Note that similar increases in blood volume are produced by venous occlusion (blue double-arrows) and arm lowering (green double-arrows). Much smaller effects are produced by venous occlusion when performed in the dependent arm (orange double-arrows). Vertical dashed lines indicate the beginning of cuff inflation and deflation. (**b**) Effect of muscle contraction on blood volume indices in a representative recording. Recordings are collected from the calf muscles during isometric plantar flexion of the ankle, the subject laying prone. From top to bottom, the electromyographic signal (EMG, measured over the distal portion of the soleus muscle), the force (measured at the forefoot) and THI signals form two probes, one located over an intramuscular soleus vein (blue) and one over a more distal portion of the same muscle (red). Note the different magnitude of contraction-related changes of THI at the two sites.

1. In Fig. 5 the execution of two occlusive stimuli in sequence produced markedly different responses in the tHb_V_ signal: while on the first occlusion (left) tHb_V_ reveals a normal progressive increase in blood volume, on the second occlusion the increase surprisingly takes place in two steps. Analysis of the US recording clarifies the phenomenon, showing that beside the basilic vein, one brachial vein also appears into the sample volume of the probe as exhibited in Supplementary Video S1 (10.6084/m9.figshare.14694270). These veins are located at 2.4 and 5.1 mm of depth and, thus, within the sampling depth of the NIRS (about 2 cm, given the inter-optode distance of 4 cm). They have been separately tracked and the time course of their change in size is also reported; it can be observed that they simultaneously dilate in response to the first but not to the second venous occlusion. The delayed dilation of the basilic vein (green trace) explain the discontinuous increase of tHb_V_. Also note the relatively small increase exhibited by tHb_M_ compared to tHb_V_.
2. In Fig. 6, the response to two subsequent rapid occlusions is shown, in a different subject. It can be observed that (1) both tHb_V_ and THI_V_, but not tHb_M_ and THI_M_ exhibit a regular oscillatory pattern (of respiratory origin) before and after the occlusion, the same oscillation being clearly visible on the BV cross-sectional area and (2) upon release of the first occlusion (but not the second) tHb_V_ and THI_V_, but not tHb_M_ and THI_M_, remain elevated with respect to the pre-occlusion levels, which again mirrors the pattern exhibited by the BV cross-sectional area. Notably, a respiratory pattern was never observed on NIRS signals collected from muscle.
3. We mentioned above that, surprisingly, in 4 subjects no THI increase was observed in the muscle, during the rapid venous occlusion. Aiming to discern whether the effect was related to individual characteristics or to the specific position of the probe on the muscle belly, we have reinvestigated the issue. We observed that small displacements (in the order of 1 cm) of the NIRS probe could revert the THI_M_ response into a prominent increase (Fig. 7). In one case this was attributed to the presence of a small vein at a depth of 23 mm, hardly detectable by B-mode US imaging but visible through Color-Doppler imaging at the time of cuff deflation.
4. Although it is well known that large changes in venous size may take place during postural changes, a representative recording is here reported to show that similar effects are produced by venous occlusion and displacement from an independent to a dependent position (Fig. 8a). A single NIRS probe is here located over the BV. It can be observed that, both tHb and THI reveal a blood volume fall when the arm is raised up and an equivalent increase when it is returned to the dependent position. Venous occlusion performed in the independent position produces comparable effects, while a smaller response is observed in the dependent position.
5. In order to show that the influence on NIRS blood volume indexes by large veins also concerns muscle contractions, a specific experimental set-up was designed. Given that it is difficult to identify large veins in the biceps muscle, this recording was performed in calf muscles, during a light isometric plantar flexion of the ankle. One NIRS probe was located over a large vein detected by US in the soleus muscle, while the other probe was located in an adjacent area of the same muscle, free of visible veins. The results are presented in Fig. 8b, whereby EMG and Force tracings document the duration and extent of the contraction. Note that the probe located over the vein detected a large decrease in blood volume during the contraction, compared to the other probe. Simultaneous US monitoring of the vein confirmed the complete vein collapse during the contraction.

## Discussion

In the present study, several points of evidence were gathered against the commonly held concept that large blood vessels do not affect the NIRS measurement. By comparing the blood volume measurements collected from the biceps muscle belly and from over the large basilic vein during venous occlusion, we showed that the increase in NIRS signal in the latter case (1) is 1.7 to 4 times larger, (2) exhibits a larger convexity and (3) is better correlated with the increase in size of the basilic vein. In addition, a number of representative recordings was shown to further describe the clear dependence of the NIRS variables from the underlying large vein(s).

In 1994, Mancini et al.^7^ stated in the Introduction of their pioneering study that, due to the high Hb concentration in the blood, emitted NIR photons were unlikely to emerge from arteries and veins larger than 1 mm in diameter, and thus NIR light absorption changes would primarily depend on smaller vessels. Although this issue was not specifically investigated in the study, this paper is highly cited to support the assumption that the presence of large vessels in the NIRS sample volume can be *tout-court* neglected. However, based on the reasoning presented in Fig. 1a and the present experimental data, this assumption appears to be incorrect in all conditions in which the size of these large vessels changes. While in arteries relevant size changes are not common, in veins they are very frequent and may easily occur due to alteration of the intravascular as well as of the extravascular pressure. However, since these factors affect large as well as small veins, the contribution of large veins is difficult to discriminate and, to our knowledge, has not been previously investigated, although some hints can be found in the literature.

Several studies pointed out that the NIRS measurement yields different results depending on probe location. For example, the impact of probe location was specifically addressed in healthy subjects by comparing the changes in tissue oxygenation in thenar and forearm muscles in response to a vascular occlusion test^20^. A significantly faster O_2_ desaturation in thenar than in forearm muscles was attributed to different anatomical structures (muscle and subdermal tissues) at the two sites. Among these, the large veins in the forearm could have played a role, considering that during arterial occlusion redistribution of the blood from arteries to veins takes place. In fact, it was also reported that the forearm exhibits a larger volume decrease than the thenar in response to lower-body negative pressure^21^. While a different sensitivity of the two regions to hypovolemic stress is possible, the higher blood volume decrease in the forearm could also be explained by a higher density of large veins undergoing emptying in the forearm than in thenar.

Interestingly, in two studies a NIRS probe was intentionally placed over visible (superficial) veins, for comparing the signals with a probe located on a virtually vein-free area^11,15^. In the first study, aimed to infer venous saturation from respiratory oscillations in venous blood volume, the authors noticed that blood volume signals collected from the vastus medialis over a visible vein had larger respiratory oscillations and provided more reliable estimation of venous oxygen saturation than signals from a vein free area of the vastus lateralis muscle^11^. This suggests that the veins may have exerted a direct influence on NIRS measurements. This interpretation is supported by the present data: respiratory oscillations were only observed on signals collected from the vein and were mirrored by respiratory changes in BV cross-sectional area (Fig. 6). The influence of superficial veins on NIRS blood volume indicators was also recently pointed out with a different approach: it was observed that the peripheral (remote) cutaneous hyperaemia, obtained by selective hand warming, produced a significant tHb increase at the forearm, with a tendency to larger effects if the probe was placed over a superficial visible vein than over a vein-free area^15^. Since no heat-induced hyperaemia was observed at the forearm, those results indicated that increased venous outflow from the hand could be detected by NIRS at the forearm.

### Subjects exhibiting a different pattern of response

In a relevant fraction of subjects (4 out of 11) the expected blood volume increase in response to venous occlusion was regularly recorded at the site over the basilic vein (tHb_V_ and THI_V_), and in the superficial (cutaneous) layers over the muscle belly (tHb_M_ increased) but not in the deeper muscle tissue (THI_M_ exhibited a negligible increase or even a decrease during cuff inflation). However, by reinvestigating the relevant subjects we showed that a prominent THI_M_ increase could be detected after slight displacement of the probe over the muscle belly (Fig. 7).

This means that the possible lack of response of THI_M_ to venous occlusion does not necessarily reflect a characteristic of that subject or of that muscle but may simply depend of the specific probe position with respect to deep veins.

Along the same line, NIRS signals may exhibit different patterns of response to muscle contraction, i.e., marked reduction if a large vein is included in the sample volume or negligible changes otherwise as shown in the example recordings of Fig. 8b.

Whether the likelihood of including a large vein in the sample volume depends on muscle mass, training, sex or other factors remains to be investigated.

### Beer–Lambert vs. spatially-resolved spectroscopy

Spatially resolved spectroscopy is known to focus the measurement in depth, thereby being less affected than the standard Beer–Lambert spectroscopy by hemodynamic changes taking place in superficial tissue layers, as has been reported for both the cerebral and muscle investigations by our and other groups^10,15,22–24^. We here observed remarkably similar responses of tHb (BL) and THI (SRS) blood volume indicators from the probe placed on the BV, in all subjects, and from the muscle probe from most subjects, which is only apparently in contrast with our previous study on the forearm, in which only tHb and not THI was affected by increased venous return from the hand^15^. In fact, in that case only the superficial venous circulation was affected, while in the present case the BV was located at a depth of about 1 cm, i.e., in a range relevant to both BL and SRS methodologies. Along the same line, the saturation of THI_V_, initially observed in two subjects and later prevented by slight probe displacement, is reasonably attributable to excessive light absorption by the vein during venous occlusion.

### Implications

NIR spectroscopy is employed to investigate several issues of physiological and clinical interest^4,25–27^. We here consider two applications that are concerned by the present results.

Assessment of *vascular compliance* by NIRS is a possible alternative to the classical strain-gauge plethysmography. Initially proposed by Binzoni et al.^28^, this measurement is based on relating the increase in blood volume (ΔtHb) to the increase in venous pressure as may be obtained by performing a Head-Up Tilt Test^28,29^, or by proximal venous occlusions^30,31^. With the present study we show that the presence of a large vein in the NIRS sample volume may magnify the blood volume change by a factor of 1.7 to 4 times (Figs. 2, 4) and alter the estimated compliance by a similar amount (Fig. 3). Although the present study was not designed to assess vascular compliance, qualitative volume-pressure and compliance curves could be obtained from the signals recorded during slow cuff deflation, highlighting the different responses at the two measuring sites (Fig. 3). The present findings are in agreement with the concept that small intramuscular vessels may have a limited dilatory capacity as compared to large extra muscular vessels. By monitoring the size of deep vessels by magnetic resonance imaging during venous occlusion, it was observed that dilatation of deep veins accounted for most of the limb volume increase, particularly at low pressures (up to 40 mmHg)^32,33^. Similar observations were reported by de Groot 2005^34^: the authors observed that, during venous occlusion, deep veins exhibited early dilatation and could reach a plateau in just 30 s, while standard plethysmography could require up to 2–3 min. These data fit with the present observation of larger and earlier changes and larger convexity of the NIRS tracings collected on the vein as compared to the muscle.

NIRS has also been used to provide indirect measurement of *muscle blood flow*. The procedure consists of assessing the blood volume increase (e.g., by means of tHb) in response to a rapid venous occlusion at sub-diastolic pressure (60–80 mmHg)^3,35,36^. Blood flow is then estimated as the initial slope of the volume curve, e.g., considered in the first 1–4 cardiac cycles^37,38^. Although in some study a good reliability of blood flow measurement has been reported^3^, other studies evidenced some variability, possibly dependent on the location of the NIRS probes^38^. By examining the initial slope of the volume responses to the rapid 40-mmHg venous occlusion of Fig. 4a, it is clear that the presence of a large vein in the sampling volume may lead to a dramatic overestimation of muscle blood flow. Conversely, low vascular compliance of muscles deprived of large veins may lead to its underestimation.

### Limitations

Our NIRS device did not account for possible changes in the differential path-length factor. In addition, even the slow venous occlusion protocol might have been too short lasting to ensure matching between venous and cuff pressures from the very beginning of cuff deflation, as required to correctly estimate volume-pressure curves (Fig. 3). However, these approximations are unlikely to explain the consistent differences between BV and muscle NIRS monitoring observed in the different conditions. The presence of subcutaneous tissue layer is also considered to affect the tissue measurement^3^, however, given small and similar thickness of cutaneous and subcutaneous tissue layers at the two measurement sites they are unlikely responsible for the observed results.

## Conclusions

For the first time NIRS blood volume indexes were collected over a large deep vein while simultaneously monitoring its size and were compared to indexes collected from the muscle belly. The results contradict the dogma that the NIRS measurement is unaffected by large vessels and confirm the hypothesis that the increase in size of large vessels determines considerable increases in NIRS blood volume indexes. Unlike superficial veins, size changes in deep veins affect Beer-Lambert as well as spatially resolved indexes. These results bear implication in all conditions affecting the vein transmural pressure, including exercise and postural changes, as well as the response to manoeuvres such as external compressions and venous occlusion.

## Supplementary Information

Supplementary Legends.

Supplementary Video S1.

## Data Availability

The datasets generated during and/or analysed during the current study are available from the corresponding author on reasonable request.

## Abbreviations

BL: Beer–Lambert
BV: Basilic vein
HHb: Deoxy-haemoglobin
nAUC: Normalized area under the curve
NIR: Near-infrared light
NIRS: Near-infrared light spectroscopy
O_2_Hb: Oxy-haemoglobin
SRS: Spatially-resolved spectroscopy
tHb: Total haemoglobin
THI: Total haemoglobin index
US: Ultrasound
