# Supplementary material for: Evidence that large vessels do affect near infrared spectroscopy
Source: Sci Rep. 2022 Feb 9;12:2155. doi: 10.1038/s41598-022-05863-y (PMC8828816; doi:10.1038/s41598-022-05863-y)
Supplement: Supplementary file 1 — Supplementary Legends. [file 41598_2022_5863_MOESM1_ESM.docx]

Original B-mode ultrasound video clip from a subject showing the responses to two subsequent short-lasting venous occlusions (30 and 60 mmHg, respectively), leading to the dilation of the brachial vein and the basilic vein. The veins are initially collapsed, being the arm raised above heart level. Note the delayed dilatation of the basilic vein during the second occluding stimulus.
